# A clinical case of recurrent episcleritis as the initial manifestation of granulomatosis with polyangiitis

**DOI:** 10.22336/rjo.2021.76

**Published:** 2021

**Authors:** Andra Carmina Ciotoracu, Monica Gabriela Dimăncescu, Traian Costin Mitulescu, Claudia Iuliana Haralambie, Ana-Maria Iorga, Constantin Busuioc, Denisa Predețeanu

**Affiliations:** * Department of Rheumatology and Internal Medicine, “Sfânta Maria” Clinical Hospital, Bucharest, Romania; ** Department of Ophthalmology, “Sfânta Maria” Clinical Hospital, Bucharest, Romania; *** Department of Ophthalmology, University Emergency Hospital, Bucharest, Romania; **** Sanador Medical Clinic, Bucharest, Romania; ***** Department of Pathology, “Prof. Dr. Matei Balș” National Institute of Infectious Diseases, Bucharest, Romania; ****** “Carol Davila” University of Medicine and Pharmacy, Bucharest, Romania

**Keywords:** episcleritis, granulomatosis with polyangiitis, kidney biopsy, nasal biopsy, cyclophosphamide, glucocorticoids

## Abstract

Granulomatosis with polyangiitis (GPA) is a type of small-sized blood vessel vasculitis that predominantly affects the upper airways, lungs and kidneys and associates with the presence of anti-neutrophil cytoplasmic antibodies (ANCA). Nevertheless, any organ of the body can be affected by GPA, including the eye. Occasionally, ocular involvement can be the initial manifestation, thus representing an essential clue for the physician in the early diagnosis of the disease.

We present the case of a 53-year-old woman in whom recurrent episcleritis was the first sign of a multisystem disease. All further investigations led to the final diagnosis of GPA. The remission induction therapy chosen by the rheumatologist consisted of intravenous cyclophosphamide (CP) and methylprednisolone pulse-therapy, followed by oral glucocorticoids (GC). Based on the favorable clinical and paraclinical evolution, induction therapy was replaced by remission maintenance therapy. Azathioprine (AZA) was initiated and oral GC were continued, with dose tapering. Complete remission of episcleritis was observed.

**Abbreviations:** GPA = granulomatosis with polyangiitis, EGPA = eosinophilic granulomatosis with polyangiitis, MPA = Microscopic polyangiitis, ANCA = Anti-neutrophil cytoplasmic antibodies, c-ANCA = ANCA to proteinase-3, p-ANCA = ANCA to myeloperoxidase, ELISAs = antigen-specific enzyme-linked immunosorbent assays, ENT = ear, nose, throat, CP = cyclophosphamide, NSAIDs = nonsteroidal anti-inflammatory drugs, AZA = azathioprine, GC = glucocorticoids

## Introduction

Granulomatosis with polyangiitis (GPA), formerly known as Wegener’s granulomatosis, is a rare systemic non-infectious granulomatous disease, causing endothelial inflammation and tissue damage in small-sized blood vessels throughout the body. GPA is associated with the presence of anti-neutrophil cytoplasmic antibodies (ANCA) and, together with eosinophilic granulomatosis with polyangiitis (EGPA) and microscopic polyangiitis (MPA), is included in a group of disorders called ANCA-associated vasculitides. General signs such as fatigue, weight loss, fever can often accompany specific organ involvement. In addition to the ear, nose, throat (ENT), pulmonary and renal signs, frequently encountered in patients with GPA, ocular involvement should be recognized as a possible early manifestation of the disease [**1**,**2**]. Any structure of the eye can be affected by the pathogenic process, including the episclera, as it was depicted in the following case report. This paper’s aim was to emphasize the importance of recognizing recurrent episcleritis as an early sign of GPA.

## Case report

**Fig. 1:**
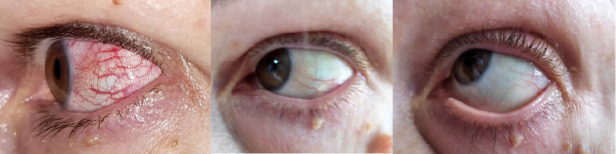
Left eye episcleritis; different stages of evolution under treatment

We present the case of a 53-year-old woman in whom ocular manifestations gave an essential clue to the presence of a systemic disease. The symptomatology of the patient had an abrupt onset and it was characterized by discomfort and watering of the left eye. No vision impairment was associated. Physical examination performed by an ophthalmologist, by using a slit-lamp, showed red episcleral discoloration, without any involvement of the sclera, thus confirming the diagnosis of episcleritis (**Fig. 1**). 

Along with a second episode of episcleritis, a more extensive evaluation was initiated in order to determine if these signs and symptoms were an early-stage presentation of a systemic disease. In one-year timeframe, the patient had a total of 5 episodes of unilateral episcleritis. The recommended treatment consisted of artificial tears and oral nonsteroidal anti-inflammatory drugs (NSAIDs). Basic blood tests, which included a complete blood count, serum chemistry profile and acute phase reactants, were performed and found to be normal. Additional serologic tests such as rheumatoid factor, antinuclear antibodies, and antibodies to cyclic citrullinated peptides were negative. Complement C3, C4 and C1q were also in normal range. ANCA using antigen-specific enzyme-linked immunosorbent assays (ELISAs) were positive for ANCA to proteinase-3 (c-ANCA) and negative for ANCA to myeloperoxidase (p-ANCA). The antibody titer of c-ANCA was 175 UI/ml (normal value < 6.7 UI/ml). Chest radiography and abdominal ultrasound were normal. Serum creatinine was normal, but repeated urinalysis with microscopic examination of the urine sediment found inconstant hematuria. Protein excretion in a 24-hour urine collection varied between 300 and 500 mg/24h. 

**Fig. 2:**
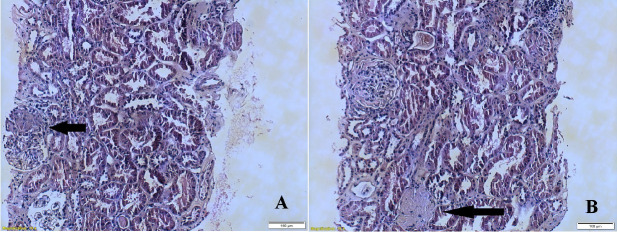
Renal biopsy. A. Renal parenchyma with glomerular mesangial cell proliferation (black arrow). B. Renal parenchyma with global glomerulosclerosis (black arrow)

The patient was also evaluated in the Nephrology Department where renal biopsy was performed. The histopathology report comprised the analysis of 16 glomeruli and described various types of lesions (with diffuse mesangial, focal extracapillary and focal segmental endocapillary patterns, one glomerulus with global glomerulosclerosis, tubular atrophy) consistent of a proliferative glomerulonephritis (**Fig. 2**). Anti-glomerular basement membrane antibodies were negative.

As the patient started developing myalgia and arthralgia of multiple joints (shoulders, elbows, knees, ankles, small joints of the hands and pain of the sacroiliac joints), further investigations were performed. The new values of inflammatory markers were approximately 2 to 3 times higher than the reference value of the laboratory (C-reactive protein 12 mg/l, erythrocyte sedimentation rate 99mm/h). Musculoskeletal ultrasound of the hands revealed tenosynovitis of both flexors and extensors of the fingers. HLA-B27 was absent and serum creatine kinase level was normal.

**Fig. 3:**
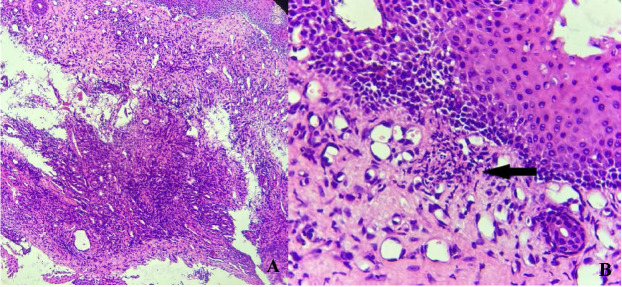
Nasal mucosa. A. Inflammatory cell infiltrate (lymphocytes, neutrophils, histiocytes) of the lamina propria (10x). B. Subepithelial infiltrate of neutrophils and red blood cells (black arrow) – suggestive of vasculitis (40x)

Bloody nasal discharge was another sign present in our patient that pointed to a systemic disease. The ENT examination was followed by a computed tomography of the sinuses with a normal result. Then, a nasal biopsy was requested, revealing inflammatory cell infiltrate (lymphocytes, neutrophils, histiocytes) of the lamina propria. Subepithelial infiltrate of neutrophils and red blood cells was also present, being suggestive of vasculitis (**Fig. 3**). Even though the histopathological report was not pathognomonic for a specific disease, the result was useful in the process of establishing the final diagnosis.

**Fig. 4:**
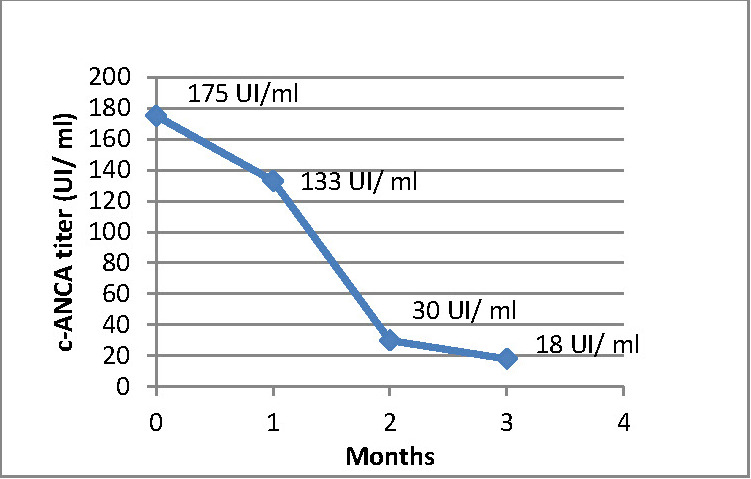
c-ANCA titer evolution during patient follow-up

Based on the clinical presentation and on all the paraclinical investigations, the diagnosis of GPA (previously known as Wegener’s granulomatosis) was established with a calculated Birmingham Vasculitis Activity Score of 18/63. The remission induction therapy was initiated. The patient had an organ-threatening type of disease, as shown by the renal biopsy, thus a remission induction regimen consisting of glucocorticoids (GC) in combination with monthly intravenous cyclophosphamide (CP) was chosen. Intravenous methylprednisolone was initially administered for 3 days (total dose over 3 days of 1500 mg) and followed by daily oral prednisone (60 mg/day) with a dose tapering regimen. Associated to GC treatment, 1000 mg of intravenous CP was also administered. Close monitoring of the patient with blood tests (complete blood count, erythrocyte sedimentation rate, C-reactive protein, serum creatinine, electrolytes) and urinalysis with microscopic examination of the urinary sediment were performed 2 weeks following infusion and at each follow-up visit. c-ANCA titer was also consistently measured, with a progressive decrease (133UI/ml, 30UI/ml, and 18UI/ml) compared to the initial value (175 UI/ml) (**Fig. 4**). After 3 months, once clinical remission was attained, CP was replaced by the remission maintenance therapy consisting of azathioprine (AZA) along with oral GC with progressive dose reduction.

During the follow-up visits, a good clinical evolution of the patient was observed with remission of episcleritis and without other symptoms. 

## Discussion

Ocular involvement can be encountered in patients diagnosed with GPA and it encompasses a large variety of signs and symptoms as it can affect any structure of the eye. The reported prevalence varies from 23 to 58% and it can be the initial feature of the disease or it can accompany other systemic manifestations [**3**-**5**]. The complex pathogenic pathways responsible for the granulomatous and vasculitis processes of GPA, which involve B and T lymphocytes as well as ANCA autoantibodies, are still not fully understood. Although rarely performed, histopathological examination of the ocular tissue can reveal inflammatory cells infiltrates consisting of plasma cells, eosinophils, neutrophils along with granulomatous inflammation, vasculitis, and collagen necrosis [**3**,**6**]. 

The episclera, a layer of tissue that lies between the conjunctiva and sclera, can be affected by the pathogenic process of GPA. In contrast to the sclera, which is considered an avascular structure, the episclera is vascularized by the superficial and deep vascular plexi. In episcleritis, which involves the superficial vascular plexi, the inflammation causes increased vascular permeability and vasodilation, resulting in a prominent appearance of these blood vessels in clinical examination [**7**].

Episcleritis and scleritis are between the most frequent ocular manifestations associated with GPA [**4**,**5**,**8**]. Episcleritis can express as diffuse inflammation, as in the case of our patient, with discomfort and mild pain, or as a localized nodular process, which generally has a longer disease course and produces more pain. In addition to pain, which is generally less pronounced than in scleritis, photophobia, ocular redness and epiphora can be present. Moreover, contrary to scleritis, episcleritis does not cause ocular visual impairment [**9**]. Episcleritis and scleritis can coexist with subsequent vasculitis that also affects the deep vascular plexi and leads to necrosis of the scleral tissue along with exposure of the choroid [**8**].

Besides GPA, episcleritis can be caused by other systemic inflammatory diseases, such as rheumatoid arthritis, systemic lupus erythematosus and inflammatory bowel disease [**10**-**12**].

Treatment approach is guided by the activity and extent of the disease, which is also reflected by specific assessment tools such as the Vasculitis Damage Index and Birmingham Vasculitis Activity Score [**13**,**14**]. In the situation of organ-threatening GPA, remission induction regimen consists of GC in association with CP or rituximab. Once the remission is attained, the remission induction therapy is followed by remission maintenance therapy [**1**]. This case report described a generalized and active type of GPA, in which ocular involvement was associated with musculoskeletal, nasal, and renal disease. The latter was considered organ-threatening and thus, required more aggressive systemic therapy with CP and methylprednisolone pulse-therapy followed by oral GC. Once remission was attained, CP was replaced by AZA. Although current data does not correlate ANCA titer with disease activity, some clinicians choose to measure it when monitoring the disease [**15**]. In the case of our patient, we observed a progressive reduction of the c-ANCA titer once treatment was initiated (**Fig. 4**). Systemic medication was effective in the remission of the organ-threatening renal signs as well as of the ocular disease. Improvement was observed over the subsequent weeks with no recurrence of episcleritis, which in this case was the early manifestation of GPA.

## Conclusion

Recurrent episcleritis can be the initial manifestation of a systemic disease such as GPA. The physician should always carefully asses the patient by obtaining a complete medical history and by searching for other associated signs and symptoms. Other severe, vision-threating complications that can be encountered in GPA should be excluded before establishing the diagnosis of episcleritis. 

The management of episcleritis secondary to GPA is challenging, as topical and oral NSAIDs can be insufficient. As described in our case report, the induction and maintenance of remission therapy of the generalized disease was also effective on the ocular involvement with complete remission of episcleritis.

Patients diagnosed with GPA often require multidisciplinary approach depending on the extent of the disease. A good collaboration between rheumatologist and physicians with other areas of expertise such as ophthalmologists, nephrologists and ENT specialists is essential for improving patient care.

**Conflict of Interest statement**

Authors state no conflict of interest.

**Informed Consent and Human and Animal Rights statement**

An informed consent was obtained from the patient included in the Case Report.

**Authorization for the use of human subjects**

Ethical approval: The research related to human use complies with all the relevant national regulations, institutional policies, is in accordance with the tenets of the Helsinki Declaration, and has been approved by the review board of “Sfânta Maria” Clinical Hospital, Bucharest, Romania.

**Acknowledgements**

None.

**Sources of Funding**

None.

**Disclosures**

None.
